# Exploring value creation in a virtual community of practice: a framework analysis for knowledge and skills development among primary care professionals

**DOI:** 10.1186/s12909-024-05061-6

**Published:** 2024-02-07

**Authors:** Débora Koatz, Alezandra Torres-Castaño, Cristina Salrach-Arnau, Lilisbeth Perestelo-Pérez, Vanesa Ramos-García, Ana Isabel González-González, Valeria Pacheco-Huergo, Ana Toledo-Chávarri, Himar González-Pacheco, Carola Orrego

**Affiliations:** 1 Avedis Donabedian Research Institute, Universitat Autònoma de Barcelona, Barcelona, Spain; 2 Canary Islands Health Research Institute Foundation (FIISC), Tenerife, Spain; 3 Evaluation Unit of the Canary Islands Health Service (SESCS), Tenerife, Spain; 4 Innovation and International Research Unit, Directorate-General for Research and Education, Madrid Health Ministry, Madrid, Spain; 5 Research Institute of University Hospital Gregorio Marañón, Madrid, Spain; 6 Centro de Atención Primaria Turó-Vilapicina, Instituto Catalán de la Salud, Barcelona, Spain; 7 Chronicity, Primary Care and Health Promotion Research Network (RICAPPS-RICORS), Madrid, Spain

**Keywords:** Patient empowerment, Community of practice, Value creation, Lifelong education

## Abstract

**Background:**

Healthcare professionals traditional education reflects constraints to face the complex needs of people with chronic diseases in primary care settings. Since more innovative and practical solutions are required, Virtual Community of Practices (vCoP) seem to better respond to learning updates, improving professional and organizational knowledge. However, little is known about the value created in vCoPs as social learning environments. The objective of this project was to explore the value creation process of a gamified vCoP (“e-mpodera vCoP”) aimed at improving the knowledge and attitudes of primary healthcare professionals (PCPs) (nurses and general practitioners) to the empowerment of people with chronic conditions.

**Methods:**

A framework analysis assessed the value creation process using a mixed methods approach. The framework provided awareness about knowledge and usefulness in a learning community through five cycles: (1) immediate value, (2) potential value, (3) applied value, (4) realized value, and (5) reframing value. Quantitative data included vCoP analytics such as logins, contributions, points, badges, and performance metrics. Qualitative data consisted of PCPs’ forum contributions from Madrid, Catalonia, and Canary Islands over 14 months.

**Results:**

A total of 185 PCPs had access to the e-mpodera vCoPs. The vCoP showed the dynamic participation of 146 PCPs, along 63 content activities posted, including a total of 3,571 contributions (including text, images, links to webpages, and other files). Regarding the value creation process, the e-mpodera vCoP seems to encompass a broad spectrum of value cycles, with indicators mostly related to cycle 1 (immediate value – activities and interactions) and cycle 2 (potential value – knowledge capital); and to a lesser extent for cycle 3 (applied value – changes in practice) and for cycle 4 (realized value – performance improvement). The presence of indicators related to cycle 5 (reframing value), was minimal, due to few individual redefinitions of success.

**Conclusion:**

To reach a wider range of value possibilities, a combination of learning objectives, competence framework, challenged-based gamified platform, and pathway model of skill development seems crucial. However, additional research is required to gain clearer insights into organizational values, professionals’ lifelong educational needs in healthcare, and the long-term sustainability of performance improvement.

**Trial registration:**

ClinicalTrials.gov, NCT02757781. Registered on 02/05/2016.

**Supplementary Information:**

The online version contains supplementary material available at 10.1186/s12909-024-05061-6.

## Background

Healthcare professionals’ education is a key issue to approach the evolving demands of populations worldwide. Although, new skills, competencies, and knowledge are required to promptly respond to population needs [1], traditional educational approaches (classes, sessions, internships) have been deemed as insufficient to translate learning into changes in practice [2] Some of the reasons for this limitation include: The swift evolution of knowledge and the irruption of technologies [3]; the attitudes of health professionals towards new practices; and the complexity of the healthcare environment that influences professionals’ behaviors in lifelong learning. Additionally, many training activities are designed for a specific discipline without addressing the need to work within an interprofessional team context [3–5]. Hence, traditional healthcare education is compelled to implement a continuous professional development approach based on new simulation strategies, innovative educational methods, the development of soft and new competencies, as well as collaborative and leadership skills [6].

Furthermore, the increase of technology-based networks seamlessly integrating formal and informal learning [7] through a unique meaningful user-centered learning experience [6] propose a shift in focus on learning, which may allow new approaches to feel professionals empowered to manage their own Personal Learning Environments (PLEs) [8].

Another important shift in the field of healthcare education is related to the value of shared learning and the relationships among people who interact and exchange knowledge and experiences regarding a common domain, interest, or problem. This can be defined as a Community of Practice [9], wherein hierarchical structures are discarded in favor of information exchange, interaction, and collaboration among peers to debate and problem-solving [10]. Learning is considered a social process [11].

Lately, virtual Communities of Practices (vCoPs) have emerged as a strategic method to ensure healthcare professionals’ continuous and experiential learning, exchanging implicit knowledge, fostering innovative ideas, improving organizational performance [12], aligning with strategic objectives, and enhancing professional growth and development [10, 13].

Although vCoPs face certain challenges (geographical distribution, different cultural backgrounds; privacy, and engagement concerns), evidence shows that vCoPs can offer an informal method for professionals‘ development and they may decrease social and professional isolation [14–16]. In primary care, collaborative work and group cohesion have been associated with enhanced access and continuity of care, and improved patient care and satisfaction, among other benefits [17–19]. Healthcare professionals may use vCoPs for searching clinically relevant and good-quality information to make more informed practice decisions [20] or may empower patients and enhance the coordination of care services [21].

Nevertheless, evaluating vCoPs’ effectiveness is a complex process [22]. Some efforts were done to understand why certain vCoPs succeed in reaching these goals over time [15], and some frameworks were created to approach this question [23–27]. However, little is known about the value creation process that remains hidden by the learning exchange of these communities in the healthcare sector [13].

Considering this context, a vCoP was created as an intervention for the e-mpodera trial described elsewhere [28, 29], that tested the potential of this vCoP for improving the knowledge and attitudes of primary healthcare professionals (PCPs) to the empowerment of people with chronic conditions. The trial demonstrated that this vCoP produced a significant improvement in participant’s attitudes towards sharing information and decision with patients, but no clear changes in caring or patient activation. However, some uncertainty remains based on limitations of the study [29].

This work aims at exploring **the value creation process of the e-mpodera vCoP**, to determine to what extent it has created value for its members and to identify key factors that facilitated value creation within this vCoP.

## Methods

### Study design

A mixed method study design was conducted, using a framework [30, 31] secondary analysis [32], of the forum’s contributions of professionals participating in the vCoP of the e-mpodera trial. It involved a qualitative design [33, 34] and a descriptive quantitative approach for main platform metrics.

### Setting

The study was carried out using the total amount of forum contributions made by participants in the e-mpodera vCoP, during the intervention of the randomized cluster control trial (March 2017 to May 2018) of e-mpodera. (http://dev.epract.net/community2/e-mpodera). ClinicalTrials.gov, NCT02757781. Registered on 02/05/2016.

### Participants

As described elsewhere [29], primary care professionals (PCPs) were recruited (November 2016 – May 2017) from urban and rural primary care practices with adequate Internet connectivity to access the vCoP, located in Catalonia, Madrid, and the Canary Islands. PCPs - general practitioners (Ps) and nurses (Ns) - belonged to the Spanish National Health System participated voluntarily, after signing an informed consent. All their contributions to the e-mpodera vCoP were extracted to be analyzed in this study.

### The intervention

The e-mpodera vCoP was a gamified virtual 2.0 platform for professionals’ knowledge exchange, which offered a guided learning pathway through patient empowerment, based on a competence framework including four learning objectives and 12 core competencies [35]. The gamification elements included points, achievement goals, badges, and a leaderboard, added to interactive content (games, problem-solving cases, surveys, audiovisual materials, and other individual and collaborative tasks). Challenges or goals required specific actions (comments to report an experience, voting on comments, a survey response, sharing opinions, ideas, or resources.), and they were awarded special points and a badge to collect (Additional File 1).

**Fig. 1:**
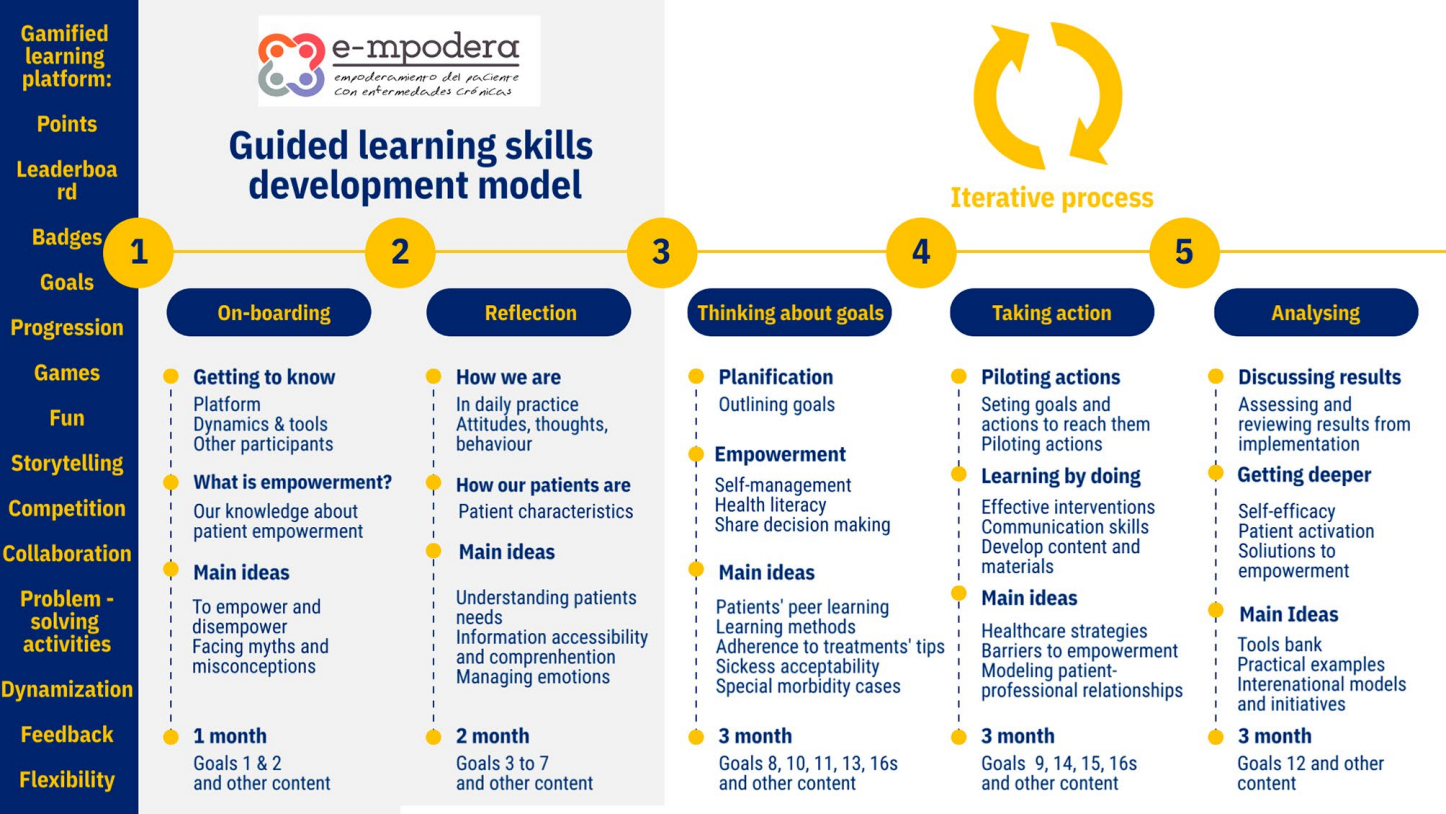
Guided learning skills development model. The guided learning skills development model depicts de learning process for professionals who participated in e-mpodera vCoP. It included five phases in a twelve-month-based intervention with different kinds of learning activities to develop specific skills and knowledge regarding empowerment. Gamification was present in the platform and activities

While there was a main pathway available, professionals were given the flexibility to follow it or chart their own course through the vCoP. New contents were added weekly, according to the e-mpodera guided learning skills development model (Fig. 1), based on prior research conducted for the e-mpodera trial [35]. Most of the activities featured dedicated forums where members engaged in conversation. A moderator actively managed the vCoP, motivated participation, identified learning gaps and needs, and answered questions.

### Data collection & analysis

Three researchers participated in the collection and analysis process of the forum’s contributions and performed extraction, coding, reviewing, analyzing, reporting, and supervising the research project.

To assess the value creation of the vCoP we performed a framework analysis, following the procedure developed by Goldsmith [31] for qualitative research in two stages:

1. The first stage, developed while the intervention was still in progress, included three steps of Goldsmith procedure: a) familiarization with the themes; b) Identification of a thematic framework, and c) Indexing data against the framework, that allowed to apply and test the indicators of the Value Creation Conceptual Framework [25] based on the first six months of contributions to the e-mpodera vCoP (March to September 2017), that were captured from the webpage and converted into PDF to be analysed by ATLAS.ti 6.1. As a result of this first moment, main framework’ indicators were adjusted to platform outputs possibilities, and a first version together with a codebook was created based on 2163 comments, in 33 forum threads, done by 144 professionals out of 163 participating.
2. In a second stage, when the intervention had already finished, the Indexing step (c) was repeated with all the e-mpodera vCoP forum contributions (March 2017 to May 2018) captured by NVivo12 R1.6 & NCapture to preserve its web format and links. Adjustments to the previous version of the codebook needed to be made to better approach the framework indicators available. Subsequently, the framework analysis procedure was completed, by steps d) Charting and e) Mapping and Interpretation.

Qualitative analysis and reporting followed the Standards for Reporting Qualitative Research (SRQR) [36]. Quantitative data were analyzed with Microsoft Excel 10 Pro.

### The framework

The Value Creation Conceptual Framework developed by Wegner et al. [25] included both qualitative and quantitative indicators. It approaches the different kinds of value raised in the learning activities. Thereby, it reveals a pattern of knowledge, usefulness, and social interaction within a learning community, based on the communication itself, rather than asking its members directly about their learning experience. This framework includes five iterative cycles: (1) Immediate value (based on activities and interaction), (2) Potential value (based on knowledge capital), (3) Applied value (focused on changes in practice), (4) Realized value (based on performance improvement), (5) Reframing value (focused on redefining success). These cycles interact and feed back to each other, reflecting the complex and dynamic nature of the learning process. This perspective assumes that, if the community does not add value at either the individual or collective level, members would be less likely to actively participate and therefore the community vanishes [25].

The final version of the framework applied is available in Table 1. The codebook and examples of quotations are described in Additional File 2.

**Table 1:** The Conceptual Framework of Value Creation for e-mpodera vCoP^1^

Conceptual framework of value creation for a virtual Community of Practice (vCoP)			
Indicators	Measures/Metrics	Codes	Definition
**Cycle 1- Immediate value: Activities and interactions**			
Level of participation	Members recruited. Logins per user, mean (SD) Users ≥ 2 logins (returning users)		
Level of activity	Users ≥ 1 comment Comments per user, mean (SD) Comments per week, mean (SD) Responses in surveys, mean (SD) Questions asked	Agreement Identification	Agreement expression and or identification with other users’ thoughts. Similar opinion.
		Asking for help or an opinion	Queries/support asked about an area of interest (explicit question) a. Ask for help or concrete query (open-ended questions). b. Question asked (closed-ended), reflective or rhetorical questions. c. Questions related platform and navigation
Level of engagement	Comments per forum (length of threads), mean (SD) Comments in goals Comments in other contents Forum threads opened by users. Users ≥ 1 point Users ≥ 1 goal (*n* = 147) Badges per user, mean (SD)		
Quality of interactions	Nested chats: First comments (level 1) Nested chats: Replicates (levels + 2)	Practice experience	Members bring experiences and problems of daily practice into the learning space. Discussion on important issues: reflections of practice, barriers, and difficulties in general. a. Problems and causes. b. Solutions applied to main barriers. c. Impact on professionals and/or patients
		Quality of response	Feedback on the quality of responses to queries. Reasons reported of the value of answers or resources shared.
		Assertive disagreement	Respectful participation when disagreement
Value of Participation		Back Reengaging	Evidence of people coming back to the community or re-engaging with the network and reasons reported.
		Fun	Evidence of fun
Networking^2^		Naming	Name someone or answer directly to his/her comment.
Collaboration	Number of writers in collaborative documents, mean (SD)		
		Collaborative initiative	Joining projects or co-authorship
		Resources and tools shared	Shared tools /resources… Shared solutions or new ways to use tools and/or resources…
Reflection		Feeling of community	References to the community itself: being a group (meta-conversations) with a common mission (learning/ empowerment)
**Cycle 2. Potential value: Knowledge capital**			
Information received	Total Products exchanged by users (images, docs, links, etc.).	Information received	New information received and reported by users
		Solution offered / Answers to questions	Answers to questions asked. New practices to solve problems (explicit).
Skills acquired		Skills, ideas, or resources to be applied	Self-reported comments related to skills acquired or resources to be used. a. Likely to be used. b. Reported to be applied
Change in perspective		Professional change expectations	Self-reported change or reflection regarding own behavior/ Getting inspired. Discussion on professional role
Confidence		Risks taken, or initiatives taken	An initiative started or risk taken by members
Level of trust		Circumstance, mistake, or failure in own practice	Self-reported difficulties, problems, or mistakes/failures from practices by members (bringing up difficult problems and failures from practice). Personal cases, not general issues.
New views of learning		Learning and leadership	Interest in learning and leadership activities
**Cycle 3. Applied Value**			
Implementation of advice, solutions, insights	Documents reported used.	Implementation	Self-reported comments on implementation of solutions, advice, insight. Ideas commented previously on the community.
Innovation in practice		Innovation insight	New ways of doing things. New perspectives. New concepts and language
Use of tools and documents to inform practice		Results	Self-reported feedback value on tools or documents used or applied. Self-reported reuse.
Use of social connections		Social Connections	Collaborative arrangements and problem-solving. Leveraging connections in the accomplishment of tasks
Innovation in systems		New Systems	Evidence of new processes and/or policies.
Transferring learning practices		Transferring practices	Use of the community or peer-to-peer processes and tools for learning in other contexts
**Cycle 4. Realized value: Performance improvement**			
Personal performance	Members with certifications (20 goals and 1000 points obtained) Members with more than 10 goals obtained		
Organizational reputation		Patient feedback^3^	Client feedback regarding empowerment reported.
Knowledge products as performances		Client outcomes	Direct delivery of knowledge products to clients (applied tools, resources to clients and outcomes)
Organizational performance		Organization satisfaction	If reported in final comments or feedback, satisfaction to organization
**Cycle 5. Reframing value: redefinition of success**			
Community aspirations		Reported new vision or learning objectives	New learning agenda. New discourse about value. New vision. Community aspirations for future
Assessment		New metrics	New metrics, assessment, or ways to evaluate. Criteria change.
Relationships with stakeholders		Relations with stakeholders	Involvement of new stakeholders. New sets of expectations Different conversation with stakeholders reported
Institutional changes		Organizational change	New strategic directions that reflect new understanding
New frameworks		New framework	New social, institutional, legal, or political systems (emerging or created)

^1^Adapted from Wegner E, Trayner B, de Laat M. Promoting and assessing value creation in communities and networks: a conceptual framework. 2011

^2^The platform doesn’t register the connections between participants, such us a user’s lists, or followers

^3^Patients were not asked directly about satisfaction or professional change

## Results

We analyzed the contributions of 146 professionals out of 185 recruited. e-mpodera vCoP participants’ characteristics are described in Table 2.

**Table 2:** Participants’ characteristics

Characteristics	Frequency
Age (years), mean (SD)	47.0 (8.5)
Sex, n (%)	
Male Female	31 (21.2%) 115 (78.8%)
Profession, n (%)	
Physicians Nurses	80 (54.8%) 66 (45.2%)
Residents tutor, n (%)	
No Yes	112 (76.7%) 34 (23.3%)
Years of experience, mean (SD)	21.7 (8.1)
Years in primary care, mean (SD)	18.0 (8.3)
Daily case load, mean (SD)	27.8 (10.7)
Years in the health centre, mean (SD)	8.2 (7.7)
SD = Standard deviation	

For 65 weeks, a total of 3571 contributions were done in the 63 forum threads of the vCoP, of which 3379 belonged to 146 participants. Two contents included surveys with 69 and 99 responses each and four collaborative documents. Quantitative results relevant to each value creation cycle are presented in Table 3. Cycles of value creation are described through indicators that progressively decrease in presence and intensity while the platform evolves over time and in stages.

**Table 3:** Quantitative results for the five cycles of value creation

Quantitative indicators	Frequency
**Cycle I. Immediate Value:** ** Level of participation**	
Members recruited	185 (8 drop-outs*)
Logins weekly-basis, mean (SD) Logins per user, mean (SD) Users ≥ 2 logins (returning users), n (%)	53.8 (32.2) 19 (29.7) 158 (85.4%)
** Level of activity**	
Users ≥ 1 comment, n (%) Comments per user, mean (SD) Comments per week, mean (SD) Responses in 2 surveys, mean (SD) Questions asked, n	146 (78.9%) 18 (21.5) 52 (47.7) 84 (21.2) 106
**Type of activity**, n (%)	
Goals Other contents	21 (33.3%) 42 (66.7%)
** Level of engagement**	
Comments per forum (length of threads), mean (SD) Comments in goals n (%) Comments in other contents, n (%) Forum threads opened by users, n (%) Users ≥ 1 points, n (%) Users ≥ 1 goal, n (%) Badges per user, mean (SD)	53.6 (63.3) 2572 (76.1%) 807 (23.9%) 4 (6.34%) 149 (80.5%) 147 (79.5%) 12.4 (8.1)
** Collaboration**	
Users writing in 4 collaborative documents, mean (SD)	16.8 (6.7)
** Quality of interactions, n (%)**	
Nested chats: First comments (level 1) Nested chats: Replicates (levels 2 to 6)	2574 (75.2%) 805 (23.8%)
**Cycle 2. Potential value**: ** Production of tools and documents to inform practice**	
Total Products exchanged by users (images, docs, links, etc.), n Images, n (%) Documents or files, n (%) Links, n (%) Solutions offered by members (to member’s questions), n Solutions offered (to problem solve challenges), n	1366 352 (25.8%) 148 (10.8%) 866 (63.4%) 48 104
**Cycle 3. Applied value**: ** Implementation of solutions, n**	
Num. shared solutions reported as implemented. Num. actions implemented (in problem-solving challenges)	11 78
**Cycle 4. Realized value**: ** Personal performance, n (%)**	
Members certified (20 goals and 1000 points required) Members with more than 10 goals	57 (38,7%) 25 (17%)

SD = Standard deviation

* Causes of drop-outs: 7 professionals argued lack of time to participate, and 1 personal reasons

### Cycle 1. Immediate value. Activities and interactions

During the initial three months of the vCoP’s launch, the onboarding process promoted a strong and immediate value creation structure. The exchange of comments allowed members to introduce themselves, discover common interests, and engage in reflections related to empowerment as the central topic.

*– I am xxx… Physician from xxx. I believe that empowering patients is fundamental and facilitates the results in the long run, improving the health of the patients, which is definitively what is pursued. (Ps_429)*

Bringing experiences and problems of daily practice into the learning space was the main activity that enriched this level. The discussion revolved around significant issues such as challenges and barriers to empowerment, possible causes, and their impact on professionals and/or patients.

*– Due to the lack of time and the care burden, we ended up giving them some guidelines that we have decided to be like this, guidelines suitable for the process, but perhaps not all suitable for the person. (Ns_349)*

Several characteristics of the activity itself may account for the high rate of returning to the vCoP (85.4%), such as having fun or meeting other professionals facing similar issues.

*– What a good time I had with the video! I still have a smile on my face;) (Ns_348)*.

*– Hello @xxx, I laughed reading your comment because everything you say has happened to me with hair and signs! (Ps_306)*

The goal-based pathway proved to be highly engaging, with 76.1% of comments concentrated in 33.3% of the goals activities. Particularly, those with entertaining actions such as sharing images of (de)empowerment and characterizing patients received a higher number of comments. Additionally, 79.5% of participants reach at least one challenge and gamification elements (points, badges, and leaderboard) likely contributed to goal orientation. Furthermore, the feedback reported showed participants’ interest and perceived usefulness of the vCoP as main values related to their participation.

*– The score motivates me. (Ns_142)*

*– Thank you very much for the contributions! Very interesting… (Ns_331)*.

*– A month has passed since my post, and I can keep on saying that patients have become more involved and that I find this method useful. (Ns_198)*

Although nested chats accounted for 23.8% of the comments, the community heavily relied on answering questions as a main action, since most activities were based on a question, and many members opted to respond directly to the main post instead of using the “replay” button.

In addition to discussing practice-related problems, some questions sought assistance or opinions: open-ended queries seeking specific advice; reflective or rhetorical questions, and inquiries related to the platform itself. A total of 106 different questions were posted by members.

*– What experiences or knowledge do you have that group education is more effective in many cases to empower patients? (Ns_331)*

*– Do all patients want that empowerment? It implies a shared responsibility. Are all patients willing to assume their share of responsibility? (Ps_267)*

*– Can someone tell me where the “Training Pills” are?” (Ns_278)*

Collaboration efforts were limited to a few initiatives with stated intentions, however notable progress was achieved when the moderator proposed specific activities (Challenges 16a, 16b, 16c and 16d).

*– I have chosen this group because we are currently carrying out an edition in our city of the Expert Caregiver Program of Catalonia, where I participate as an observer together with @I…. who is also doing this course within this same group. (Ns_139)*

Engaging in content queries, and expressing personal opinions allowed flow and nurtured a respectful environment and a feeling of community.

*– And here I do not agree with other colleagues… (Ps_141)*.

*– I would also raise the case in the same way… (Ps_174)*.

*– I hope to enjoy this experience and that we all learn a lot from each other. (Ns_243)*

### Cycle 2. Potential value: knowledge capital

This level includes connections, new concepts learned, skills and tools potentially useful for the future. Getting to know each other, relationships deepen, fostering a heightened level of trust within the community, which encouraged members to openly share not only “good practices”, but also their mistakes, challenges, professional failures, ad problem-solving skills. This reflects the personal and human value of participation and the sense of companionship of social learning (social and human capital).

*– These days I have learned something. As a result of several claims that I have received from treatment. (Ns_247)*

Deeper reflections about their own practices and expectations were done. The relationship between patients and physician, professionals’ role, and potential for bringing about change started to be discussed (reputational capital).

*– After reading the article I have learned that the problems regarding understanding and communication of information are not based on people’s minds, but in the way in which the problem to be solved is represented. (Ps_215)*

Users initiated some debates by asking for tools and calling for action.

*– What experience or knowledge do you have about group education being more effective to empower patients, in many cases? (Ns_331)*

*– Do you think it’s good to share resources? (Ps_210)*

Furthermore, solutions to concrete problems were offered, such as initiatives to share directories of resources, relevant documents, and links (interviews, surveys, infographics, etc.). A total of 48 responses aimed at addressing members’ queries were provided, primarily involving ideas and some resources, which were well appreciated by users, who expressed their interest and chances to use them. Enthusiasm for in learning activities was also expressed at this level. Other responses were solved by the moderator.

*– I think it is very applicable into day-to-day practice and I will do so. (Ps_215)*

*– The format (of the e-mpodera vCoP) is interesting, it gives rise to several options to exchange with other members of e-mpodera and learn at the same time (Ns_254)*

Certain challenges proposed a patient’s case to problem-solve (Challenge 10 and 13), resulting in 130 solutions. The iterative nature of discussions facilitated accumulative knowledge, as solutions were refined and improved throughout the thread and feedback was provided on each solution.

*– I liked this approach! However, we must inform the patient that we can initiate a therapeutic strategy, either hygienic-dietary or pharmacological measures and that we are able to substitute, replace or do both at the same time, they are not exclusive, far from it. (Ps_145)*

The content types that received the highest number of solutions were mainly problem-solving cases (Challenge 10 with 74 and 13, with 56), and those opened by users (likely due to the specific nature of queries), followed by the collaborative challenges (16a, 16b, 16c, and 16d).

A total of 1366 resources were exchanged by users, including self-created materials, research papers and files from the Internet. Products shared addressed topics of interest and provided answers to specific questions. For instance, information to share with patients, images from congresses, etc. (tangible capital). Due to platform limitations, it was not possible to track the number of downloads for each resource, unless explicitly mentioned in comments, as new information received.

*– I think the survey was excellent. I didn’t know it. I will certainly try to apply it in my work. (Ns_309)*

Confidence within the group also boosted sharing images of their work environments, ongoing projects and real situations.

The solutions to questions and specific challenges hold the potential of application in different practice context. The method and format of the activity offered could also serve as inspiration for members (learning capital).

*– The format is interesting, it gives rise to several options to exchange with other members of e-mpodera and learn at the same time (Ns_254)*

### Cycle 3. applied value: change in practice

Time played a crucial role in transforming potential capital into applied value, alongside motivation to share outcomes within the vCoP. The e-mpodera guided learning skills development model along with the moderator’s facilitation, enabled members to engage in action-oriented activities and analyze the results. Challenges 9, 10, 13, 14, and 15 exemplify this type of activity. For instance, Challenge 9 involved implementing practice improvements, assessing results after pilot testing, and expanding the scope of implementation (to be explained in Challenge 14).

*– I have piloted the experience with 2 patients, LMG* (patient name) *a 28-year-old woman and DAG* (patient name), *a 26-year-old man, both with debut asthma (Ps_215)*.

Certain practices implemented didn’t rely on previously shared information within the community, but the implementation itself fostered the opportunity to test and share as a common value. Members shared positive and negative results, enabling exploration to improve the practice they wanted to change. Sometimes, these ideas entailed novel approaches, fresh perspectives on concepts and alternative language usage, which could inspire further ideas.

*– In my view, there are “empowerable” patients and others are not. (Ps_165)*

*– As I write this, I have realized that by disempowering the patient, he relieves himself of responsibility. (Ps_157)*

Reporting results of applied resources and tools, holds a key importance within professional learning environments. As members receive this information, they gain insights that get inspired to replicate the experience, innovate, and strive for new and improved results.

*– The experience has been very interesting and the practical effects in improving medication intake in the elderly were practically immediate. (Ps_292)*

*– This experience was very negative in our pilot, but it helped us to improve in the following exercise prescription activities. Most of the patients abandoned the activity in the first sessions. (Ps_141)*

Some of the results were obtained in complex projects, where the opportunity appeared and the innovation could potentially be applied to newly implemented systems (expert-patient projects, e-consultation, etc.).

*– Patients have enthusiastically received the launch of the e-consultation for doubts and questions, and during this time I have answered 12 questions (basically related to treatment, side effects…) (Ps_215)*.

Moreover, transferring what is happening in the vCoP to other spaces entails the creation of something valuable, allowing other contexts or individuals to benefit from that learning.

*– I have been able to carry out the exercise with a co-worker, although she does not participate in e-mpodera. (Ps_182)*

*– I’m sorry, I have shared it on Facebook for my acquaintances and friends. It seems very real and very cruel! (Ps_141)*

The use of social connections made them gang together for collaboration in a task, also promoted by the e-mpodera guided learning skills development model. As a result, a series of challenges (16 A, 16B, 16C2, 16D) were proposed to prepare several workshops for patients regarding various conditions, such as obesity, dementia, ischemic heart disease and chronic obstructive pulmonary disease. These activities prompted participants to reflect on the practical use of their social learning.

*– I’m not in this group but I do congratulate you! I loved it. Very well structured. I would love to be able to do it in my health center one day!!!! (Ns_126)*

### Cycle 4. realized value: performance improvement

At this level, evidence available from the vCoP was limited since organizational aspects were not included into the intervention design. Even though, organizational performance is identified.

*– With these training sessions we have reduced the cost of glycaemic test strips, patients have improved self-control and we have introduced a behavior change in the control of DM. (Ns_113)*

Considering professionals’ performance indicators available in the vCoP, 38.77% of participants obtained a training certification (available for members with 1000 points and 20 challenges completed), while a total of 55.78% achieved 10 or more goals, which represents half of the e-mpodera activities proposed. Overall, most of the results of the experiences shared after implementing new practices can be seen as professional achievements, particularly when positive results are observed.

Professionals reported delivering knowledge products to clients, including tools and resources.

*– In total, I was able to extend the activity to 40 families, I quantified that more than 80% had consulted the Internet for information from the prenatal to preschool period. I realized that most of them did not feel safe and were even embarrassed to admit that they had consulted web pages to find out about the care of their children. This is how the directory of web pages “Together from the beginning 2.0” arises, where parents and other caregivers can find out about care during pregnancy, childbirth, the puerperium, breastfeeding, first care of the newborn, complementary feeding, etc. (Ns_348.)*

However, not all applied knowledge resulted in performance improvements that align with stakeholders’ priorities. To gather comprehensive indicators at this level, additional data from patients (feedback and satisfaction) is needed for supplementation.

### Cycle 5. reframing value: redefinition of success

This redefinition of success can occur at individual, collective, and organizational levels. Few findings of this cycle were identified, mainly referring to new assessment methods and metrics to redefine success. One example included the time distribution for actions in consultations, addressed through a survey activity.

*– Many of the patients who started exercising with us have changed their habits and continue to exercise in a group. And not just them but myself too. Before, I only took a few minutes to talk about exercise and now it is a priority part of the daily consultation. (Ps_211)*

New frameworks emerge for certain participants, regarding patient expert programs, that were fully implemented in some facilities, while in others were discovered as an effective approach for empowerment within this vCoP.

*– In our practice, groups with “the expert patient” have been incorporated. A highly recommended and effective experience. (Ps_250)*

*– The only thing that is not implanted in the day to day, is something punctual. (Ns_247)*

*– The organizational improvement level wasn’t considered in the design of the vCoP, nor in the trial. However, few specifics comments referred to organizational change implemented.*

*– A simple circuit for receiving CS procedures is provided for local clinics that, due to their size, do not have administrative professionals for customer service. (Ps_157)*

At individual level, a new framework regarding empowerment and its impact was achieved.

*– This course has made me really get down to work and I believe in it in every-day practice; empowering improves the doctor-patient relationship, the self-management of the disease and the quality of life of patients. (Ns_247)*

## Discussion

The results obtained demonstrate that e-mpodera raises four key aspects of a vCoP in health sector [24]: Active social interaction among members (Cycle 1), sharing knowledge (Cycles 1 and 2), creating knowledge (Cycles 2 and 3), and building identity (all cycles) [37–39].

Over time, the vCoP showed progress in the different cycles of value creation, aligning with the core principles for cultivating vCoPs [40]. The design of evolution is reflected in the guided learning pathway that progressively shows the growing interactivity through the activities proposed. From individual to collaborative, from daily practice to specific problems, from queries to new approaches. However, the level of activity and participation gradually decreased over time. Preliminary results in the first stage of the value creation assessment showed that 64% of contributions occurred within the first 6 months of the intervention, gathering almost 88% of participants. This could be attributed to holiday breaks. Although the vCoP remained open throughout, overall participation decreased in these periods.

Moreover, a rhythm was established through challenges and other content that weekly fed the community, following other of the core principles for cultivating vCoPs. Furthermore, the gamified [41] elements may explain the higher participation in the challenges activities (76.1% of total comments). This “game” approach for learning purposes highlights the entertainment as another important key factor for an engaging vCoP, providing suitable participation incentives [42].

A respectful environment is relevant to create a positive feeling of shared community [43], and reinforces the group mission of learning and task orientation. This creates a virtuous circle wherein the feeling of community enhances trust and strengthens the identity of belonging, boosting communication and socialization [11]. It is worth mentioning the horizontal and equal interdisciplinary exchange shown by professionals in different positions based on sharing their experiences equally and searching for mutual support, despite the hierarchical structure traditionally present in training in clinical settings [44].

Additionally, familiarity, trust, and commitment among members were essential for building a risk-free environment where various levels of participation were welcome [16] (reading, voting, commenting, sharing, working together, etc.). Nevertheless, the value derived from peripheral participation could be hard to detect without specific indicators [45].

The idea that the community has a specific goal to achieve (to improve the knowledge and skills of PCPs for the empowerment of patients with chronic diseases) seemed to motivate the contributions of the participants [15, 46].

The moderator played a crucial role in ensuring an effective community environment [47], with a mission to encourage participation, identify members’ needs and knowledge gaps, provide relevant content and manage content exchanged [46, 48]. In addition, the moderator of e-mpodera, supported by an interdisciplinary research team, followed the skills development model previously created to align with the trial’s purpose [35]. This shared leadership showed to be positive in building trust and encourage participation. However, it might have discouraged members to bringing up new topics aside the content’s threads proposed, as only 5 debates were opened for questions and opinions. Therefore, boosting natural leadership and supporting roles among members, is needed for future sustainability [12].

In addition to the shared explicit knowledge (documents, care plans, protocols, etc.,), it seems relevant to focus on the tacit knowledge that emerges from the reflections on the practice itself, and the narratives shared among professionals (opinions, success and failure stories, solutions to problems, and feedback on strategies) [49].

These findings are also consistent with previous research conducted in higher education, which also employed the value creation framework with differences in research methods [50, 51].

## Conclusions

The value creation conceptual framework proved to be a valid approach for assessing the value created within e-mpodera vCoP. The evidence of immediate value is clearly shown through the rich exchange of comments raised and the interactivity with the platform’s contents.

Potential value encompassed a deeper commitment and trust in the collective learning environment. This led a wide range of capital: human, social, tangible, reputational and learning capital) based mainly on ideas, strategies, and resources towards patient’s empowerment.

Social interaction became a factor of learning [11] through professionals’ questions, feedback exchange (proposals, recommendations, suggestions, or solutions) and shared ideas willing to be applied.

The applied value was mainly given by the opportunity to translate the capital knowledge that flowed within the vCoP into actions, boosted by targeted challenges. Identifying changes in practice was limited to user-reported experiences and outcomes. However, capturing the impact of improvement on patients required a safe and supportive environment, adequate time for evolution, and specific opportunities provided mainly by the moderator. Evidence of reframed value was reduced to the individual level as a new framework for empowerment and novel criteria and metrics to consider it.

The combination of a learning objectives and competence framework, the challenged-based gamified vCoP along with the guided skills development model, seems to be key in unlocking a broad spectrum of value possibilities, particularly in terms of knowledge application and performance improvement. The moderator played a central role in facilitating knowledge exchange and providing confidence for building a safe space for collaboration, experimentation, inquiry, and guidance-seeking. Further research is needed to gain deeper insights regarding organizational values, healthcare professionals’ lifelong educational needs and long-term sustainability of performance improvement.

### Limitations

Several limitations are noted in this work. As a secondary analysis of an intervention within a randomized control trial, this work is limited by the type and availability of data sources requiring adaptation to align to the original framework indicators, which in some cases was not possible. Firstly, the unavailability of data on thread reads and downloaded documents hampers the assessment of value creation among peripheral participants. Moreover, a lack of a common set of platform metrics in similar studies [45, 51] discourage comparison with other studies, particularly in active participation indicators. Secondly, the design and structure of the content-based platform didn’t allow researchers to clearly identify connections among users, establish a reputation history and analyze leadership potential for sustaining the vCoP. Thirdly, considering the value creation framework analysis, value creation reported directly from participants needs to be supplemented with data from other sources, such as interviews and focus groups. It is worth mentioning that what is mostly reported in forums is an intention but is not likely to assume that it means changes in real practice. In this case, this additional data collection was not conducted to avoid interfering with the trial’s development. Moreover, other variables should be considered to contrast the observed activity with learning outcomes and professionals’ performance analysis. Fourthly, the time-limited intervention could have limited gathering evidence of cycles four and five. Finally, satisfaction and self-reported values of the learning activity would have provided important inputs for the overall intervention if done, to explore its potential sustainability in an open environment.

### Electronic supplementary material

Below is the link to the electronic supplementary material.

Supplementary Material 1

Supplementary Material 2

## Data Availability

- The datasets generated and/or analysed during the current study are not publicly available due to individuals’ data protection regulations but are available from the corresponding author on reasonable request.

## Abbreviations

vCoP: Virtual Community of Practice
PCP: Primary Healthcare Professionals
Ps: General practitioners
Ns: Nurses
PLE: Personal learning environment
DM: Diabetes Miellitus
