# Supplementary material for: Exploring value creation in a virtual community of practice: a framework analysis for knowledge and skills development among primary care professionals
Source: BMC Med Educ. 2024 Feb 7;24:121. doi: 10.1186/s12909-024-05061-6 (PMC10848396; doi:10.1186/s12909-024-05061-6)
Supplement: Supplementary file 1 — Supplementary Material 1 [file 12909_2024_5061_MOESM1_ESM.docx]

**Additional File 1:**

**Examples of activities proposed in challenges of the e-mpodera vCoP**

| Challenges | Activity proposed | Description |
| --- | --- | --- |
| 1 | Introduce yourself and complete your user profile | Starting participation in the vCoP by self-introducing and explaining topics of interest and previous knowledge regarding patient empowerment. |
| 2 | Do we work together? | Watch a funny video and reflect on the roles of patients and doctors. |
| 3 | Access to information | Discussion on the possibility of patients navigating the Internet to search for clinical information. |
| 8 | Planning empowerment actions | Planning empowerment actions regarding a topic of interest: Choose objectives, indicators, and measures to assess those objectives, tasks to implement the goals and specific material. Finally, share your plan with the vCoP. |
| 9 | Implementing the planned actions | Piloting actions planned in the previous challenge. |
| 10 | Case: Do you remember Sarah? | To develop an interview considering a previous case and choose the better interview voting. |
| 13 | Case: Trying or not? | To develop an interview considering a previous case and choose the better interview voting. |
| 14 | Practicing the Share Decision Making | To put in practice the interview done in Challenge 13 with a colleague, first as a patient, then as a professional. |
| 15 | Collaborative challenge: empowerment group workshop! | Select an option for creating a group workshop to empower patients regarding:  Group A: Obesity, diabetes mellitus  Group B: Hypertension, ischemic heart disease, heart failure  Group C: Chronic obstructive pulmonary disease, asthma c  Group D: Elderly patient, fall prevention, stroke, dementia, caregiver care.  The group will be created through vCoP members willing to participate in each workshop. |
| 16 (a,b,c,d) | Group A: Obesity, diabetes mellitus  Group B: Hypertension, ischemic heart disease, heart failure  Group C: Chronic obstructive pulmonary disease, asthma c  Group D: Elderly patient, fall prevention, stroke, dementia, caregiver care. | Each group has a collaborative document to share contents, and ideas, and elaborate a group workshop for patients regarding each topic. for the collaborative task.  When finalizing, vCoP members will vote for the better workshops developed. |
