# Supplementary material for: Exploring value creation in a virtual community of practice: a framework analysis for knowledge and skills development among primary care professionals
Source: BMC Med Educ. 2024 Feb 7;24:121. doi: 10.1186/s12909-024-05061-6 (PMC10848396; doi:10.1186/s12909-024-05061-6)
Supplement: Supplementary file 2 — Supplementary Material 2 [file 12909_2024_5061_MOESM2_ESM.docx]

| Additional File 2: Codebook and quotations | | | |
| --- | --- | --- | --- |
| Cycle 1 Indicators | **Codes** | **Definition** | **Example quotations** |
| Level of activity | 1.Agreement Identification | Agreement expression and or identification with other user’s thoughts. Similar opinion. | - *Completely agree with the previous opinions… (Ps_114)* - *I totally agree with your answer. (Ns_142)* - *I would also raise the case in the same way… (Ps_174)* |
|  | 2.Asking for help or opinion | Queries/support asked about an area of interest (explicit question):  2.a Ask for help or concrete query (open-ended questions).  2.b Question asked (closed-ended), reflective or rhetorical questions.  2.c Questions related platform and navigation | *-- 2.a What experiences or knowledge do you have that group education is more effective in many cases to empower patients? (Ns_331)*   - *I would like to know experiences of virtual workshops…* - 2.b *Do all patients want that empowerment? It implies a shared responsibility. Are all patients willing to assume their share of responsibility? (Ps_267)* - *What do you think, comrades? (Ns_353)* - 2.c *Can someone tell me where the "Training Pills" are?” (Ns_278)* |
| Quality of interactions | 3.Practice experience | Members bring experiences and problems of daily practice into the learning space. Discussion on important issues: reflections of practice, barriers, and difficulties in general.  3.a Problems and causes.  3.b Solutions applied to main barriers.  3.c Impact on professionals and/or patients. | - 3.a *Yes, the computer is a very clear communication barrier that interferes with our interviews. (Ps_215)* - 3.a *Due to the lack of time and the care pressure, we ended up giving them some guidelines that we have decided to be like this, guidelines suitable for the process, but perhaps not all suitable for the person. (Ns_349)* - 3b*.I use a lot of graphic representations that I make at the time, drawings or I give data in figures to reinforce my information. I usually give them the graphics and I think they like them and take it more seriously (Ps_437)* - 3b. Searching for strategies to share within the screen has two advantages: it improves the patient's information and brings us closer to the communication level. *(Ps_215)* - 3c. *An informed patient, committed to his/her care and who takes responsibility and participates in decision-making, is a patient who will undoubtedly respond better to his/her illness. (Ps_429)* - 3c*. It is important that they are curious and that they understand their disease, if they* *are able to look for information that will help us "to take it better" we can help a lot in controlling the disease. (Ps_195)* |
|  | 4.Quality of response | Feedback on quality of responses to queries. Reasons reported of value of answers or resources shared. | - *Little by little these concepts are elucidated, thanks for the clarifications (Ps_164)* - *Thank you very much for the contributions! Very interesting… (Ns_331)* - *I see that the proposed tools can be of comfortable use, in Spanish, and appropriate for the health problems exposed. (Ps_211)* - *I didn't know about the survey either and I find it useful. (Ps_197)* |
|  | 5.Assertive disagreement | Respectful participation when disagreement. | - *When activities are carried out, and here I do not agree with other colleagues… (Ps_141)* - *I have not felt identified (PS_162)* - *Hi, I don't quite agree. The healthcare professional is stand-up, and the other three people are sitting and listening… (Ns_156)* |
| Value of participation | 6.Back Reengaging | Evidence of people coming back to community or reengaging with the network and reasons reported. | - *Excuse me... I've been away without Wi-Fi for 15 days! (Ps_210)* - *A month has passed since my post and I can keep on saying that patients have become more involved and that I find this method useful. (Ns_198)* - *After rereading the previous comments about the labels, I am left with a reflection: Behind each label there is a person, a patient with a hidden problem that we should discover. That could be our challenge! (Ps_306)* |
|  | 7.Fun | Evidence of fun | - *hahaha it happened to me too! I had to repeat it again to focus on the message...(Ns_331)* - *What a good time I had with the video! I still have a smile on my face ;) (Ns_348)* - *Hello @xxx, I laughed reading your comment because everything you say has happened to me with hair and signs! (Ps_306)* |
| Networking^[[1]](#footnote-1)^ | 8.Naming | Name someone or answer directly to his/her comment. | - *@P…: I think your contribution is great…* - *Totally agree with @f…) (Ns_266)* - *I also believe, like @J…@, that it is a good tool to teach patients different scales...(Ps_182)* |
| Collaboration | 9.Collaborative initiative | Joining projects or co-authorship | - *Good afternoon, I'm joining the group* (for the collaborative task) *a little late. I have made some contributions in blue… (Ns_149)* - *I have chosen this group because we are currently carrying out an edition in our city of the Expert Caregiver Program of Catalonia, where I participate as an observer together with @I…. who is also doing this course within this same group. (Ns_139)* |
|  | 10.Resources and tools shared | Shared tools /resources… Shared solutions or new ways to use tools and/or resources... | - *I leave you the link of an APP that the Fundación Tomillo has made for the elaboration of healthy recipes… (Ns_430)* - *I leave the link for more information: (Ns_348)* |
| Reflection | 11.Feeling of community | References to the community itself and being a group (meta-conversations) | - *I hope to enjoy this experience and that we all learn a lot from each other. (Ns_243)* - *Forums are the main collective intelligence tool. (Ps_210)* |
| Cycle 2 Indicators | **Codes** | **Definition** | **Quotations** |
| Information received | 12.Information received | New information received reported by users. | - *I was unaware of this app. Thanks for sharing. All knowledge is good from my point of view. (Ns_247)* - *With all this of e-mpodera I am asking myself many questions… (Ns_366)* |
|  | 13.Solution offered / Answers to questions | Answers to questions asked.  New practices to solve problems (explicit). | - *To address non-adherence to treatment, I propose, as a first step, to learn more about the problem. I have found two articles that can bring us some light closer to your understanding. (Ns 430)* - *Visually, infographics and gamification can help the user to improve and strengthen the knowledge they should have about their pathology. (Ns 156)* - *I find it (your interview) very complete and structured, but as other colleagues said, it is too extensive to address all the points in a single visit. It could be developed in different visits. (Ps_214)* |
| Skills acquired | 14.Skills, ideas, or resources to be applied | Self-reported comments related skills acquired or resources to be used.   1. Likely to be use. 2. Reported to be applied. | - *15a. I love your workshop. (…) Congratulations!!!! (Ps_209)* - *15a. I like it, it would be good to put it into practice (Ns_115)* - *15b. I think the survey was excellent. I didn't know it. I will certainly try to apply it in my work. (Ns_309)* - *15b. I think it is very applicable into day-to-day practice and I will do so. (Ps_215)* |
| Change in perspective | 15. Professional change expectations | Self-reported change or reflection regarding own behaviour/ Getting inspired.  Discussion on professional role. | - *Very interesting and instructive. It has made me reflect and visualize situations of daily consultation. (Ns_247)* - *After all this information and reflecting on it, I realized that sometimes we don't say it so easily and we believe that with percentages it is understood when it is not. (Ns_198)* - *After reading the article I have learned that the problems in the understanding and communication of information do not reside in the minds of people, but in the way in which the problem to be solved is represented. (Ps_215)* |
| Confidence | 16.Risks taken, or initiatives taken | Initiative started or risk taken by members. | - *I make some proposals with the intention of helping to achieve the objectives of the workshop, tell me what you think:(Ps_211)* - *I'm going to break the ice. Here's a draft, to get started. (Ns_170)* |
| Level of trust | 17.Circumstances, mistake, or failure in own practice | Self-reported difficulties, problems, or mistakes/failures from practices by members (bringing up difficult problems and failures from practice). Personal cases, not general issues. | - *These days I have learned something. As a result of several claims that I have received from treatment. (Ns_247)* - *Once a patient of mine changed the physician and told him: despite being a good doctor, and explaining everything with me, she failed. (Ps_110)* |
| New views of learning | 18. Learning and leadership | Interest in learning and leadership activities. | - *I believe that it is essential to incorporate training on this subject in university education (Ps_189)* - *The format is interesting, it gives rise to several options to exchange with other members of e-mpodera and learn at the same time (Ns_254)* |
| Cycle 3 Indicators | **Codes** | **Definition** | **Quotations** |
| Implementation of advice, solutions, insights | 19. Implementation | Self-reported comments on implementation of solutions, advice, insight. Ideas commented previously on the community. | - *As a professional, I have offered several options and together, we have evaluated the benefits and disadvantages, so we have tried to reassure and gain confidence in the decisions made. (Ps_145)* - *I have piloted the experience with 2 patients, LMG* (patient name) *a 28-year-old woman and DAG* (patient name)*, a 26-year-old man, both with debut asthma (Ps_215)* |
| Innovation in practice | 20. Innovation insight | New ways of doing things. New perspectives. New concepts and language. | - *Your contribution is interesting when commenting on the possibility of contacting Diabetes associations. Empowering is not only an individual task but also a collective one (community-based) (Ps_145)* - *In my view, there are "empowerable" patients and others are not. (Ps_165)* - *As I write this, I have realized that by disempowering the patient, he relieves himself of responsibility. (Ps_157)* |
| Use of tools and documents to inform practice | 21. Results | Self-reported feedback value on tools or documents used or applied.  Self-reported reuse. | - *The experience has been very interesting and the practical effects in improving medication intake in the elderly were practically immediate. (Ps_292)* - *This experience was very negative in our pilot, but it helped us to improve in the following exercise prescription activities. Most of the patients abandoned the activity in the first sessions. (Ps_141)* - *This patient had never received advice on physical activity. I installed Google fit for him and he saw that I can actually monitor him, and he also checks it... He lost 4 kilos in a month. Especially since he had a moderate depression, that we de-dramatized together, which has also improved with physical activity. (Ps_210)* |
| Use of social connections | 22. Social Connections | Collaborative arrangements. Leveraging connections in the accomplishments of tasks | - I'm not in this group but I do congratulate you! I loved it. Very well structured. I would love to be able to do it in my health center one day!!!! *(Ns_126)* - Whenever you want! You know that a project with you will be a pleasure! *(Ps_141)* |
| Innovation in systems | 23. New Systems | Evidence of new processes and/or policies. | - Patients have enthusiastically received the launch of the e-consultation for doubts and questions, and during this time I have answered 12 questions (basically related to treatment, side effects...) *(Ps_215)* |
| Transferring learning practices | 24. Transferring practices | Use of the community or peer-to-peer processes and tools for learning in other contexts. | - I have been able to carry out the exercise with a co-worker, although she does not participate in e-mpodera. *(Ps_182)* - I'm sorry, I have shared it on Facebook for my acquaintances and friends. It seems very real and very cruel! *(Ps_141)* - I have put it on my YouTube channel, in the e-mpodera video list!!! *(Ps_210)* |
| Cycle 4: Indicators | **Codes** | **Definition** | **Quotations** |
| Organizational  reputation | 25. Patient feedback^[[2]](#footnote-2)^ | Client feedback regarding empowerment reported. | - One day a patient commented to me in a very respectful manner, that he considered me a good professional, but I was very cold and serious in the dialogue. I was surprised, my consideration was totally the opposite: accessible, empathetic. Given this situation, I considered asking different patients who answered the same. Since these answers I changed my attitude toward them. *(Ns_251)* |
| Knowledge products as performances | 26. Client outcomes | Direct delivery of knowledge products to clients (applied tools, resources to clients and outcomes). | - *In total, I was able to extend the activity to 40 families, I quantified that more than 80% had consulted the Internet for information from the prenatal to preschool period. I realized that most of them did not feel safe and were even embarrassed to admit that they had consulted web pages to find out about the care of their children. This is how the directory of web pages "Together from the beginning 2.0" arises, where parents and other caregivers can find out about care during pregnancy, childbirth, the puerperium, breastfeeding, first care of the newborn, complementary feeding, etc. (Ns_348)* |
| Organizational performance | 27. Organization satisfaction | If reported in final comments or feedback, satisfaction to organization. | - *With these training sessions we have reduced the cost of glycaemic test strips, patients have improved self-control and we have introduced a behaviour change in the control of Diabetes Miellitus (DM) (Ns_113)* |
| Cycle 5:  Indicators | **Codes** | **Definition** | **Quotations** |
| Community aspirations | 28. Reported new vision or learning objectives | New learning agenda. New discourse about value. New vision. Community aspirations for future. | - *It has allowed me to share information and reflect on ideas, as well as to become aware of the need to empower.* |
| Institutional changes | 29. Organizational change | New strategic directions that reflect new understanding | - *A simple circuit for receiving procedures for “My health folder (CS)” is provided for local clinics that, due to their size, do not have administrative professionals for customer service. (Ps_157)* |
| Assessment | 30. New metrics | New metrics, assessment, or ways to evaluate. Criteria change. | - *Many of the patients who started exercising with us have changed their habits and continue to exercise in a group. And not just them but myself too. Before, I only took a few minutes to talk about exercise and now it is a priority part of the daily consultation. (Ps_211)* |
| Relationships with stakeholders | 31. Stakeholders | Involvement of new stakeholders. New sets of expectations. Different conversations with stakeholders reported. | - *No quotation for this code.* |
| New frameworks | 32. New framework | New social, institutional, legal, or political systems (emerging or created). | - *In our practice, groups with "the expert patient" have been incorporated. A highly recommended and effective experience. (Ps_250)* - *To empower you must dialogue, allow participation, resolve contradictions... and dedicate more time, time that we think we don't have. But as shown in the video, the greater the empowerment, the lower the frequency of consultations and the more time we will have. And most importantly, our patients will have a better quality of life. (Ns-167)* - *This course has made me really get down to work and I believe in it in everyday practice; empowering improves the doctor-patient relationship, the self-management of the disease and the quality of life of patients. (Ps_210)* |

1. The platform doesn’t register the connections between participants, such us a user’s lists, or followers. [↑](#footnote-ref-1)
2. Patients were not asked directly about satisfaction or professional change.Ps: general practitioner

   Ns: Nurse

   DM: Diabetes Mellitus

   CS: My Health Folder [↑](#footnote-ref-2)
